# 

**Published:** 1874-01

**Authors:** 


### Contents of January Number.

### ORIGINAL DEPARTMENT.

ART. I.—Hints on the Treatment of Fractures of the lower extremeties. By J. F. Woods, M. D. .... 201

ART. II.—Malignant Disease of the Uterus and Vagina. By H. D. Vosburgh, M. D. .... 207

ART. III.—Glasses called "London Smoke" are better for shielding the eyes from too intense light. Translated from Annals D'Oculistique. By F. W. Abbott, M. D. .... 210

### MISCELLANEOUS.

Croton Chloral Hydrate, .... 214

Experimental Researches in Cerebral Pathology, .... 215

A New Solvent for Phosphorus, .... 216

A Circular to the Medical Profession, .... 218

A Practical Sphygmograph, .... 219

Case of Strychnine Poisoning, .... 221

Ovariotomy by Enucleation; Drainage, .... 223

Immediate Compression of the Common Iliac, .... 237

Ovariotomy by Enucleation, .... 229

### EDITORIAL DEPARTMENT.

Another Death from Anæsthesia, .... 230

Obituary.—Prof. Sandford Eastman, .... 232

Dr. S. W. Butler, .... 235

Appropriations for the U. S. A. Medical Museum and Library, .... 235

Meeting of State Medical Society—New Journal—Re-appointment—Notice—Dictionaries—Hard on Coroners, .... 236

Alumni Association of Albany Medical College, .... 237

Books Reviewed:

Lectures on Clinical Medicine. By A. Trousseau, .... 237

Treatise on Diseases and Injuries of the Eye. By Dr. Carl Stellwag, .... 238

Physicians Visiting List, .... 238

Physicians Hand Book, .... 238

A System of Midwifery. By Wm. Lieshman, M. D., .... 239

A Manual of Midwifery. By Dr. Karl Schrœder, .... 239

Books and Pamphlets Received .... 240

